# Infecciones, hospitalizaciones y mortalidad por COVID-19 en Navarra entre febrero de 2020 y septiembre de 2022

**DOI:** 10.23938/ASSN.1044

**Published:** 2023-08-16

**Authors:** Itziar Casado, Manuel García Cenoz, Nerea Egüés, Cristina Burgui, Iván Martínez-Baz, Jesús Castilla

**Affiliations:** 1 Instituto de Salud Pública y Laboral de Navarra Pamplona España; 2 CIBER Epidemiología y Salud Pública (CIBERESP) España; 3 Instituto de Investigación Sanitaria de Navarra (IdiSNA) España

**Keywords:** COVID-19, Pandemia, Mortalidad, Vigilancia epidemiológica, Vacunación del COVID-19, COVID-19, Pandemic, Mortality, Epidemiological surveillance, COVID-19 vaccination

## Abstract

**Fundamento:**

El SARS-CoV-2 circuló ininterrumpidamente en España durante el período comprendido entre febrero de 2020 y septiembre de 2022. Este estudio analiza su repercusión en las infecciones, hospitalizaciones y defunciones en Navarra.

**Métodos:**

A partir de la vigilancia epidemiológica reforzada y de los resultados de encuestas sero-epidemiológicas, se han analizado las infecciones, hospitalizaciones y defunciones por COVID-19 en función de la cobertura vacunal y otras medidas preventivas aplicadas durante el período del estudio.

**Resultados:**

Se confirmaron 295.424 personas con COVID-19 (45% de la población navarra), 8.594 requirieron ingreso hospitalario (1,3%), 832 ingresaron en unidades de cuidados intensivos (1,3‰) y 1.725 fallecieron (2,6‰). Durante la primera onda pandémica, en pocas semanas se registraron 1.934 hospitalizaciones y 529 defunciones por COVID-19 confirmado; dicha cifra se redujo significativamente tras el confinamiento domiciliario. Hasta octubre de 2021, la circulación del SARS-CoV-2 estuvo modulada por las medidas preventivas no farmacológicas. La posterior relajación de las mismas dio paso a una amplia circulación de la variante ómicron, triplicando el número de casos registrados hasta entonces. La alta cobertura vacunal frente a la COVID-19 introdujo cambios decisivos en su epidemiología, reduciendo la proporción de casos que requirieron hospitalización, ingreso en unidades de cuidados intensivos y fallecimientos a menos del 2%, 0,1% y 0,5%, respectivamente.

**Conclusiones:**

El confinamiento domiciliario inicial y las medidas preventivas no farmacológicas contuvieron la circulación del SARS-CoV-2 hasta extenderse la vacunación, con la cual se logró una reducción decisiva en la gravedad y letalidad de la COVID-19.

## Abstract (en)

**Background:**

Between February 2020 and September 2022, SARS-CoV-2 has circulated uninterruptedly throughout Spain. This study analyses COVID-19 infections, hospitalizations, and deaths in Navarre.

**Methods:**

Enhanced epidemiological surveillance and results of seroepidemiological surveys were used to analyze COVID-19 infections, hospitalizations, and deaths, based on the vaccination coverage and other preventive measures applied from February 2020 to September 2022.

**Results:**

A total of 295,424 COVID-19 cases were confirmed (45% of the population in Navarre); 8,594 required hospital admission (1.3%), 832 were admitted to intensive care units (1.3‰) and 1,725 died (2.6‰). Over the first wave of the pandemic, there were 1,934 hospitalizations and 529 deaths from confirmed COVID-19 cases over a few weeks; these figures dropped rapidly following lockdown. Until October 2021, SARS-CoV-2 circulation was modulated by non-pharmacological preventive measures. The subsequent relaxation of these measures led to a wide circulation of the omicron variant, increasing the number of cases by three-fold. The high vaccination coverage against COVID-19 introduced decisive changes in the epidemiology of the disease, reducing to less than 2%, 0.1%, and 0.5% the cases requiring hospitalization, intensive care unit admission, or that died, respectively.

**Conclusions:**

Initial lockdown and non-pharmacological preventive measures helped control SARS-CoV-2 transmission until vaccination was extended. Vaccination achieved a decisive reduction of the COVID-19 severity and lethality.

## INTRODUCCIÓN

En diciembre de 2019 se detectaron en Wuhan (China) los primeros casos de una nueva enfermedad respiratoria grave, y en enero de 2020 se identificó un nuevo coronavirus, el SARS-CoV-2, como causa de esta enfermedad, denominada COVID-19. Esta infección se propagó rápidamente, detectándose el primer caso en España el 30 de enero de 2020 y en Navarra el 28 de febrero. El 11 de marzo la Organización Mundial de la Salud declaró el COVID-19 como pandemia^1^.

Aunque en la mayoría de los casos la COVID-19 cursaba de forma leve, en algunos pacientes evolucionaba a cuadros de neumonía que podía llegar a ser mortal^2^^-^^4^. Los aspectos fundamentales de la transmisión tardaron en conocerse con detalle, lo que dificultó la implantación temprana de medidas preventivas específicas eficaces^2^^-^^5^.

Durante los dos primeros meses de la pandemia, la disponibilidad de pruebas diagnósticas fue limitada, destinándose principalmente a los pacientes graves hospitalizados^6^^,^^7^. Además, el periodo de varios días que a veces se observaba entre la infección y la hospitalización retrasó la detección de la progresión de la pandemia.

Cuando hubo constancia de la enorme propagación que la infección estaba alcanzando en la población española, fue imperiosa la implantación de un confinamiento domiciliario de la población. El estado de alarma que incluyó este confinamiento se promulgó el 14 de marzo, y finalizó el 26 de junio de 2020, tras una progresiva desescalada de las medidas preventivas^8^^-^^10^. Entre mayo y diciembre de 2020 se aplicaron diferentes intervenciones, tratando de buscar el punto de equilibrio que permitiese mantener las actividades esenciales y productivas de la sociedad, conteniendo la tendencia del SARS-CoV-2 a una rápida propagación.

A lo largo de la pandemia se sucedieron diversas variantes del SARS-CoV-2 que presentaban características diferentes de transmisibilidad, virulencia, letalidad y evasión parcial a anticuerpos generados frente a variantes previas^11^^,^^12^. Desde enero de 2021 empezó a haber una proporción creciente de la población que había recibido alguna dosis de vacuna frente a la COVID-19^13^. La actividad social fue aumentando hasta alcanzar condiciones próximas a la normalidad en el invierno de 2021-2022.

El presente estudio tiene por objetivo describir y caracterizar el curso de la pandemia de COVID-19 en Navarra en términos de número de infecciones, hospitalizaciones y defunciones.

## MATERIAL Y MÉTODOS

El presente estudio descriptivo analizó información del sistema de vigilancia reforzada de casos confirmados de COVID-19 en Navarra^13^^,^^14^. Como fuentes complementarias se utilizaron los estudios seroepidemiológicos de la infección por SARS-CoV-2 realizados en España en 2020 (ENE-COVID)^7^^,^^15^ y en Navarra en 2022^16^, el sistema de información sobre mortalidad diaria (MoMo)^17^, y el registro de vacunaciones de Navarra.

Se consideró el primer diagnóstico de COVID-19 en cada persona realizado entre febrero de 2020 y septiembre de 2022. No se consideraron reinfecciones porque su vigilancia planteó situaciones de valoración compleja y porque su peso en las infecciones y en los casos graves fue pequeño durante el periodo de estudio.

La vigilancia reforzada de casos de COVID-19 se basó en la notificación obligatoria de todos los casos confirmados en los centros sanitarios y en laboratorios públicos y privados. Durante todo el periodo de estudio, la confirmación de casos se realizó mediante la técnica de reacción en cadena de la polimerasa (PCR), que detectó la presencia de material genético del virus en la muestra obtenida de la nasofaringe del paciente. La disponibilidad limitada de reactivos hasta abril de 2020 ocasionó un sub-diagnóstico considerable de infecciones leves y, en los primeros momentos, también un sub-diagnóstico de infecciones en pacientes hospitalizados y fallecidos. Sin embargo, los pacientes que ingresaron en unidades de cuidados intensivos (UCI), probablemente fueron correctamente confirmados durante toda la pandemia. Entre abril y mayo de 2020 también se consideró confirmatorio el resultado positivo a pruebas rápidas de detección de anticuerpos en pacientes que presentaban clínica sospechosa de COVID-19. Desde octubre de 2020 comenzaron a considerarse casos de COVID-19 aquellos que presentaban una prueba de antígenos positiva, y desde diciembre de 2021, se incluyeron también los resultados positivos de auto-test de antígenos notificados desde las oficinas de farmacia o por los propios pacientes, con lo que se adecuó la capacidad de detección de casos a los niveles de incidencia de cada momento.

Todos los ingresos y defunciones en pacientes confirmados con COVID-19 fueron revisados por médicos de salud pública para establecer la causalidad de la COVID-19 sobre el ingreso o la defunción. Se consideraron ingresos por COVID-19, aquellos en los que se confirmó el SARS-CoV-2 antes o durante el ingreso, siempre que el motivo del ingreso o su prolongación fuera atribuible a esta infección. El mismo criterio se aplicó a los ingresos en UCI. Se consideraron muertes por COVID-19, aquellas ocurridas en pacientes con confirmación de infección por SARS-CoV-2, en los que ésta infección pudo contribuir a la muerte, independientemente de que la defunción hubiese ocurrido durante el ingreso, en días posteriores o sin producirse ingreso. Los ingresos y defunciones en los que el papel de la COVID-19 fue dudoso, también se clasificaron como debidos a COVID-19.

Se utilizaron fuentes complementarias de información para contrastar y corregir posibles desvíos de la información de la vigilancia epidemiológica Como la incidencia de infecciones en la primera onda estuvo muy afectada por el sub-diagnóstico, las estimaciones de incidencia se corrigieron tomando como referencia las estimaciones de seroprevalencia en Navarra obtenidas en la tercera ronda del Estudio de Seroprevalencia de anticuerpos frente al SARS-CoV-2 (ENE-COVID)^7^. Los detalles de la metodología se han descrito en un estudio previo^10^.

La posible infra-detección de defunciones por COVID-19 en la primera onda pandémica se corrigió aplicando el exceso de mortalidad observado entre el 16 de marzo y el 19 de abril de 2020 en Navarra, según los datos del sistema de monitorización de la mortalidad diaria (MoMo) que recoge información de los registros civiles^10^. Para corregir la posible infra-detección del número total de personas que habían pasado la infección por SARS-CoV-2 durante el periodo de estudio, se utilizaron los resultados del Estudio Seroepidemiológico de anticuerpos frente al SARS-CoV-2 en la población de Navarra en mayo de 2022^16^.

Los datos de cobertura vacunal frente a la COVID-19 al final de cada periodo se obtuvieron del registro autonómico de vacunaciones. Los cambios en la incidencia observados se valoraron en función de la cobertura vacunal y de las medidas preventivas establecidas en cada momento.

## RESULTADOS

### Incidencia de casos confirmados de COVID-19

Hasta septiembre de 2022 se habían confirmado 295.424 personas con COVID-19, lo que supone el 45% de la población. Además, la encuesta de seroprevalencia indicó en mayo de 2022 que el 62% de la población de Navarra tenía anticuerpos de infección pasada por SARS-CoV-2, y que aproximadamente un tercio de las infecciones habrían quedado sin confirmar, lo que corrige la estimación de personas que habían pasado esta infección hasta un rango entre 420.000-480.000, lo que supone entre dos terceras y tres cuartas partes de la población (Tabla 1).

**Tabla 1:** Resumen en cifras de la pandemia de COVID-19 en Navarra, febrero de 2020 a septiembre de 2022

	N	Tasa por 1.000 habitantes
Casos confirmados	295.424	448
Número estimado de personas que han pasado la infección	420.000-480.000	620-710
Ingresos hospitalarios por COVID-19 confirmado	8.594	13
Ingresos en unidades de cuidados intensivos	832	1,3
Defunciones por COVID-19 de casos confirmados	1725	2,6
Estimación corregida de defunciones por COVID-19	1.900-1.960	2,9-3,0

**Figura 1:**
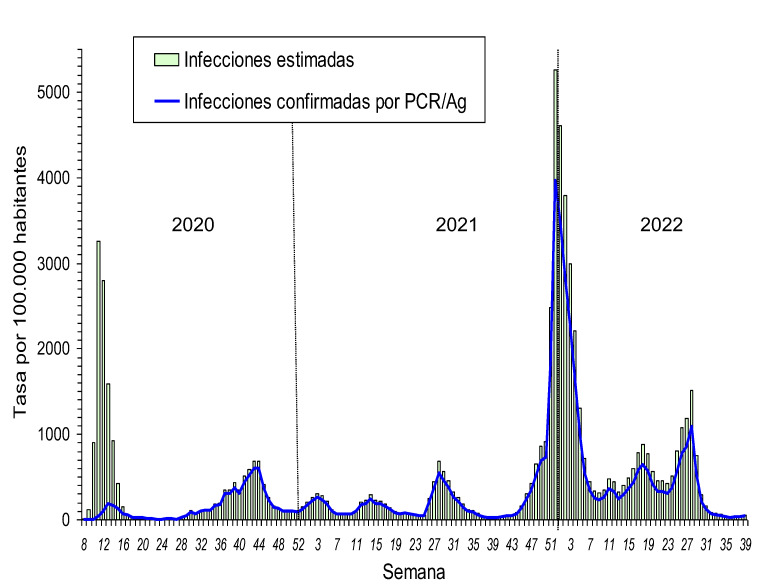
Tasa de incidencia semanal confirmada y estimada de infección por SARS-CoV-2 en Navarra por 100.000 habitantes, febrero de 2020 a septiembre de 2022.

La incidencia de casos de COVID-19 estimados con la corrección tuvo un despegue extraordinariamente abrupto en la primera quincena de marzo de 2020, y alcanzó el nivel máximo en torno a la fecha de declaración del estado de alarma (14 de marzo), momento en el que se produjo un cambio radical en la tendencia, iniciando un rápido descenso (Fig. 1).

**Figura 2:**
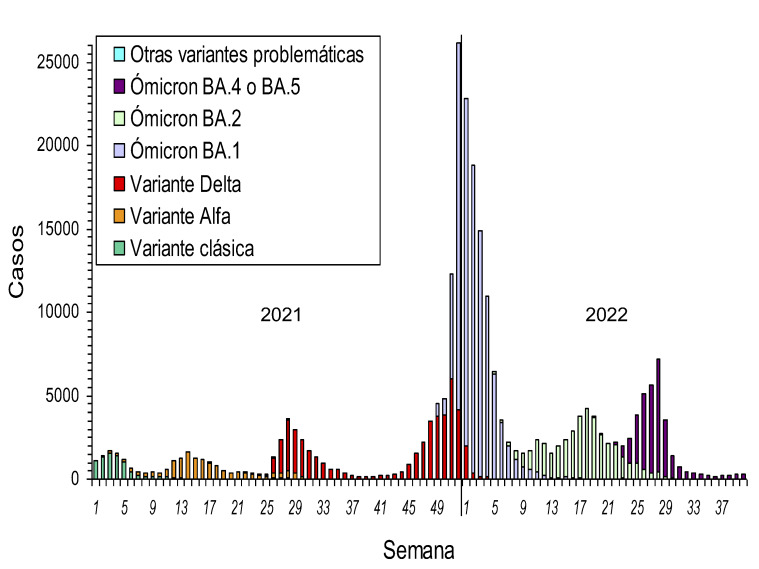
Incidencia semanal de casos de COVID-19 confirmados y su distribución en función del porcentaje estimado de cada variante, enero de 2021 a septiembre de 2022.

Entre mayo de 2020 y octubre de 2021 se sucedieron cuatro ondas epidémicas moderadas, algunas de las cuales coincidieron con la propagación de las variantes Alfa y Delta del SARS-CoV-2 (Fig. 2) y cuya remisión coincidió con la acentuación de medidas preventivas no farmacológicas que se implantaban en respuesta a cada aumento en la incidencia. Desde noviembre de 2021 se produjo una onda epidémica de grandes dimensiones asociada a la rápida propagación de la variante Ómicron y a la relajación de algunas medidas preventivas. Desde octubre de 2021 hasta septiembre de 2022 se confirmaron en Navarra 208.173 casos de COVID-19, duplicando ampliamente el número de casos registrados durante los 18 meses previos de pandemia.

**Figura 3:**
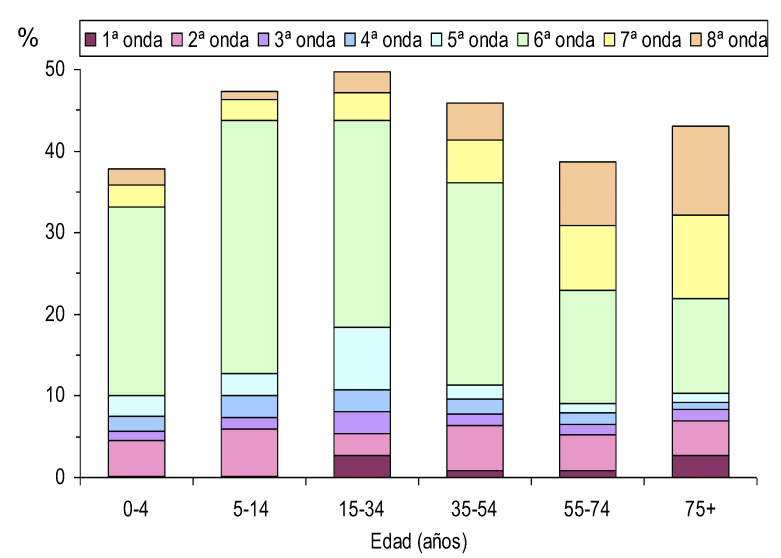
Proporción de la población con COVID-19 confirmada en las distintas ondas pandémicas según el grupo de edad, febrero de 2020 a septiembre de 2022.

A finales de septiembre de 2022, el 45% de la población de Navarra había tenido al menos un diagnóstico confirmado de COVID-19, sin grandes diferencias en función de la edad, con un rango entre el 38% en menores de 5 años y el 50% en el grupo de 35 a 54 años. Sin embargo, cada onda afectó de forma diferente a los distintos grupos de edad. La primera onda afectó especialmente a las personas mayores de 75 años, mientras que en la sexta onda la incidencia fue marcadamente menor en mayores de 55 años, lo que acabó compensándose con una mayor incidencia en las siguientes ondas (Fig. 3).

### Ingresos hospitalarios por COVID-19

**Figura 4:**
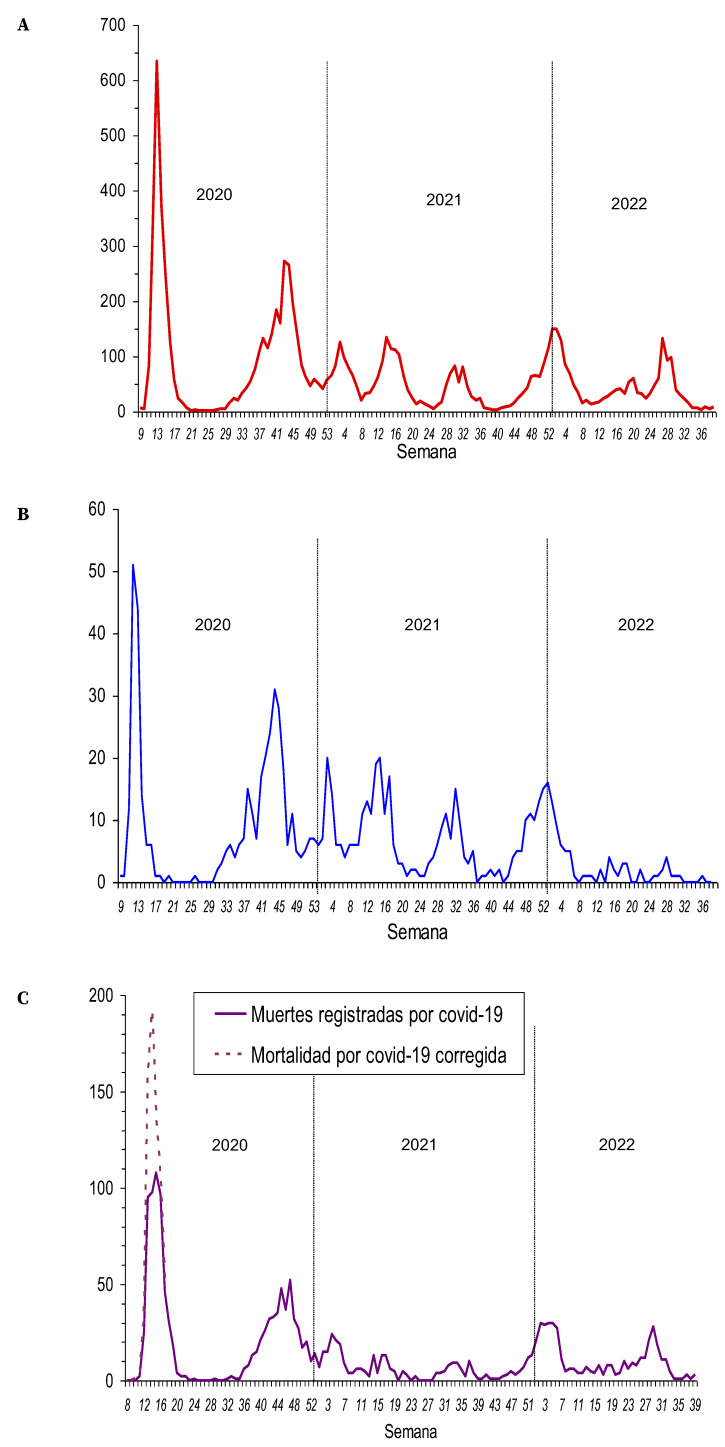
Número semanal de personas que ingresaron por COVID-19 en el hospital (**A**) y en unidades de cuidados intensivos (**B**), y que fallecieron por COVID-19 confirmado (**C**), entre febrero de 2020 y septiembre de 2022.

Hasta septiembre de 2022 se habían registrado 8.594 ingresos debidos a COVID-19 confirmado, que supusieron 13 ingresos por cada 1.000 habitantes, el 2,9% de los casos confirmados y el 2% del total de infecciones estimadas. Se registraron cifras de ingresos hospitalarios semanales excepcionalmente elevadas en la primera y segunda ondas pandémicas; en 2020 se produjeron la mitad de todos los ingresos por COVID-19. La proporción de casos que necesitó ingreso hospitalario alcanzó el 7,2% en la cuarta onda, que fue debida a la variante Alfa. A partir de la quinta onda, en el verano de 2021, la proporción de casos que requerían ingreso hospitalario fue descendiendo progresivamente (Tabla 2). Las ondas de ingresos hospitalarios por COVID-19 de 2021 y 2022 fueron moderadas, pero de forma atípica, se produjeron varias ondas a lo largo del año, incluyendo el periodo estival (Fig. 4A).

**Tabla 2:** Caracterización de las ondas epidémicas de COVID-19 en Navarra, 28 de febrero de 2020 a 2 de octubre de 2022

	Primera onda*	Segunda onda	Tercera onda	Cuarta onda	Quinta onda	Sexta onda	Séptima onda	Octava onda
Periodo	28/02 a 28/06/20	29/06 a 27/12/20	28/12/20 a 28/02/21	1/03 a 20/06/21	21/06 a 3/10/21	4/10/21 a 6/3/22	7/3 a 5/06/22	6/6 a 2/10/22
Estacionalidad	Invierno	Otoño	Invierno	Primavera	Verano	Invierno	Primavera	Verano
Periodo	28/02 a 28/06/20	29/06 a 27/12/20	28/12/20 a 28/02/21	1/03 a 20/06/21	21/06 a 3/10/21	4/10/21 a 6/3/22	7/3 a 5/06/22	6/6 a 2/10/22
Estacionalidad	Invierno	Otoño	Invierno	Primavera	Verano	Invierno	Primavera	Verano
Duración en semanas	17	26	9	16	14	22	14	16
Casos confirmados								
Nº	10.349	36.426	9284	12.452	18.740	140.157	35.933	32.083
% de la población	1,60%	5,50%	1,40%	1,90%	2,80%	21,30%	5,50%	4,90%
Nº máximo semanal	1232	3966	1691	1596	3090	26.097	4255	7188
Semana del máximo	S13/20	S43/20	S3/21	S14/21	S28/21	S52/21	S18/22	S28/22
Hospitalizaciones								
Nº	1934	2331	639	915	462	1243	454	616
% de los casos	18,80%	6,50%	6,60%	7,20%	2,50%	0,90%	1,30%	1,90%
Nº máximo semanal	635	272	125	133	79	150	61	132
Semana del máximo	S13/20	S43/20	S3/21	S14/21	S31/21	S1/22	S20/22	S27/22
Ingresos en UCI								
Nº	139	239	76	133	79	135	19	12
% de los casos	1,34%	0,66%	0,82%	1,07%	0,42%	0,10%	0,05%	0,04%
% de los ingresos	7,20%	10,30%	11,90%	14,50%	17,10%	10,90%	4,20%	1,90%
Nº máximo semanal	51	31	20	20	15	16	4	4
Semana del máximo	S12/20	S44/20	S3/21	S15/21	S32/21	S1/22	S15/22	S29/22
Defunciones por COVID								
Nº	529	440	129	87	65	250	79	146
Letalidad	5,11%	1,21%	1,39%	0,70%	0,35%	0,18%	0,22%	0,46%
Nº máximo semanal	108	52	24	14	10	32	8	28
Semana del máximo	S15/20	S47/20	S4/21	S16/21	S37/21	S2/22	S16/22	S29/22
Variante dominante	Ancestral	EU1 B.1.177	EU1 B.1.177	Alfa B.1.1.7	Delta B.1.617.2	Ómicron BA.1	Ómicron BA.2	Ómicron BA.4/5
Cobertura vacunal								
Alguna dosis	NA	NA	6,20%	55,20%	79,7	86,70%	87,40%	87,60%
Pauta completa	NA	NA	3,50%	38,60%	78,4	83,90%	85,60%	85,70%
Dosis de refuerzo	NA	NA	NA	NA	0,9	51,60%	54,20%	56,00%

*: disponibilidad limitada de pruebas diagnósticas; NA: no aplicable.

Un total de 832 ingresos tuvieron estancia en unidades de cuidados intensivos (UCI), lo que supuso 1,3 ingresos por cada 1.000 habitantes y un 0,3% de los casos de COVID-19 confirmados. La proporción de ingresos hospitalarios que necesitó ingresar en UCI alcanzó el valor máximo (17,1%) en la quinta onda, que fue debida a la variante Delta, y descendió rápidamente a partir de entonces coincidiendo con el rápido aumento de la cobertura de vacunación completa en la población adulta (Tabla 2, Fig. 4B).

### Mortalidad relacionada con COVID-19

La vigilancia reforzada registró 1.725 defunciones debidas a COVID-19 confirmado entre marzo de 2020 y septiembre de 2022, y la correspondiente tasa acumulada de mortalidad ascendió a 262 por 100.000 habitantes. En la primera onda pandémica, las muertes con confirmación de COVID-19 infra-estimaron el exceso de mortalidad semanal observado en unas 200 defunciones, aproximadamente (Fig. 4C).

Hasta la tercera onda pandémica la letalidad de los casos de COVID-19 se mantuvo por encima del 1%. A partir de entonces, coincidiendo con la extensión de la vacunación, la letalidad descendió progresivamente hasta el 0,70% en la cuarta onda, el 0,35% en la quinta y el 0,18% en la sexta (Tabla 2).

## DISCUSION

El SARS-CoV-2 circuló de forma ininterrumpida en Navarra entre marzo de 2020 y septiembre de 2022. Tras un periodo inicial marcado por parámetros de gravedad y letalidad de la infección muy preocupantes, la generalización de la vacunación frente a la COVID-19 fue seguida por una reducción importante de estos parámetros.

La transmisión tuvo un despegue abrupto en la primera quincena de marzo de 2020. Diferencias climatológicas y medioambientales explicaron en buena parte las diferencias geográficas que se observaron en esta primera onda^18^. La COVID-19 se comportó como un problema de salud excepcionalmente grave, por el gran número de casos, hospitalizaciones, ingresos en UCI y defunciones que ocasionó en pocas semanas, desbordando la capacidad del sistema sanitario. La mortalidad por COVID-19 ocasionó un exceso de la mortalidad general y un descenso de la esperanza de vida en 2020^19^. La situación se controló mediante un confinamiento domiciliario obligatorio entre marzo y abril de 2020 que consiguió en junio el retorno a niveles bajos de incidencia^10^. La encuesta de seroprevalencia ENE-COVID estimó que el 5,7% de la población de Navarra había pasado la infección durante este periodo, lo que indicaba que el SARS-CoV-2 mantenía todavía gran parte de su potencial para causar morbilidad y mortalidad en una población que en su gran mayoría no tenía inmunidad^7^.

Entre mayo y diciembre de 2020 se aplicaron diferentes intervenciones preventivas que permitieron mantener las actividades esenciales y productivas y atenuar la segunda onda del SARS-CoV-2^13^. La contención de la transmisión que se consiguió en momentos en los que todavía no se disponía de vacuna demostró la eficacia de estas medidas preventivas no farmacológicas cuando eran aplicadas correctamente^20^. Sin embargo, la gran limitación de estas medidas era la necesidad de mantenerlas indefinidamente a lo largo del tiempo, ya que cuando se relajaban, volvían a aumentar los contagios^21^.

Desde enero de 2021, una proporción creciente de la población fue vacunada frente a la COVID-19. Las vacunas demostraron inicialmente un efecto preventivo apreciable frente a los contagios y un potente efecto frente a las formas graves de la enfermedad^22^. La efectividad de la vacunación en el control de la transmisión se redujo con la llegada de las variantes Alfa y Delta. Los aumentos de incidencia que dieron lugar a varias ondas pandémicas pudieron verse favorecidos por la llegada de estas nuevas variantes que presentaban alguna ventaja para su difusión con respecto a la variante EU1^11^. Estas variantes también se asociaron a una mayor proporción de formas graves de la enfermedad en personas que no habían completado la vacunación^12^. Durante esta etapa se fue alcanzando la vacunación completa de la mayoría de la población, y las personas más vulnerables pudieron recibir una dosis de refuerzo^13^.

Desde octubre de 2021 se fue produciendo un cambio marcado en las características epidemiológicas de la COVID-19, que pasaron a asemejarse a las de otras infecciones por virus respiratorios. La virulencia y letalidad de las infecciones por SARS-CoV-2 fueron disminuyendo debido a la alta efectividad de la vacuna para reducir la gravedad^23^^,^^24^, a la elevada cobertura de vacunación que se alcanzó tempranamente en la población de Navarra, a los progresos en el manejo clínico de las personas infectadas, y a la llegada de la variante Ómicron, que se caracterizó por una menor virulencia^12^.

A la vista del descenso en la gravedad de la COVID-19, se fue relajando la aplicación de las medidas preventivas no farmacológicas, se recuperó la normalidad en la actividad productiva y docente, y aumentó la actividad social, dando oportunidad a una amplia circulación del SARS-CoV-2 en la población.

En mayo de 2022, la encuesta de seroprevalencia de anticuerpos frente al SARS-CoV-2, mostró que el 62% de la población de Navarra tenía anticuerpos anti nucleocápside, indicativos de infección pasada. Este porcentaje superaba el 80% en menores de 30 años, caía por debajo del 43% en mayores de 60 años, y hasta el 26% en mayores de 80 años. Cuatro de cada 10 personas con anticuerpos indicativos de infección pasada, no habían tenido un diagnóstico confirmado previo de COVID-19^7^.

La presencia de estos anticuerpos de infección pasada demostró ser fundamental para evitar infecciones por SARS-CoV-2 en los meses siguientes, y probablemente esto explica que las últimas ondas (séptima y octava) ocasionasen mayor incidencia en personas de mayor edad^25^. La coincidencia de la octava onda de COVID-19 con periodos de temperaturas extremadamente altas durante el verano de 2022 hizo difícil separar el efecto del calor en la mortalidad y la letalidad atribuida a la COVID-19, al haberse podido sumar ambos efectos en las mismas personas. El aparente aumento de la letalidad en las ondas séptima y octava puede explicarse por la menor confirmación de casos leves y por la mayor proporción de casos confirmados que pertenecían a los grupos de mayor edad.

Desde octubre de 2022 el diagnóstico y la vigilancia epidemiológica de la COVID-19 se focalizaron en personas mayores de 65 años y en pacientes hospitalizados o graves. Las hospitalizaciones y defunciones por COVID-19 se mantuvieron en niveles bajos, sin dar lugar a una nueva onda epidémica propiamente dicha, lo que puede interpretarse como una normalización de la situación y la desaparición de los aspectos que definieron la situación pandémica^26^.

El presente estudio presenta algunas limitaciones. Los datos de vigilancia pueden tener desviaciones con respecto a la realidad en el número de infecciones y defunciones durante la primera onda y, en menor medida, en el número de infecciones durante todo el estudio; todo ello se trató de corregir utilizando fuentes de información alternativas. Las estimaciones proporcionadas no deben considerarse datos exactos y han de interpretarse con márgenes de error. No se han aplicado métodos estadísticos por haberse trabajado con datos de toda la población, y porque los márgenes de error no dependen tanto de la potencia estadística como de la validez de las fuentes de información, no quedando este aspecto recogido en los intervalos de confianza. Este estudio solo ha considerado primeros diagnósticos; no obstante, el riesgo de una segunda infección y su gravedad tiende a ser mucho menor que en la primera, por lo que los primeros episodios captan la mayor parte de la carga de enfermedad^27^^,^^28^, aunque también pueda producirse enfermedad grave en casos de reinfección^29^. Los ingresos y defunciones en los que el papel de la COVID-19 era dudoso se clasificaron como debidos a esta causa, lo que puede haber sobreestimando algo su impacto.

En conclusión, el SARS-CoV-2 circuló de forma continua en Navarra desde 2020 hasta 2022, demostrando un enorme potencial pandémico en ausencia de medidas eficaces de control. La primera onda pandémica causó un número considerable de hospitalizaciones y defunciones por COVID-19 en pocas semanas y pudo controlarse con la implantación de un confinamiento domiciliario.

Hasta octubre de 2021, la circulación del SARS-CoV-2 estuvo muy modulada por las medidas preventivas no farmacológicas que se aplicaron. La relajación de estas medidas fue seguida por aumentos en la incidencia. La generalización de la vacunación frente a la COVID-19 introdujo cambios decisivos en la epidemiología de la enfermedad, reduciendo considerablemente la proporción de casos que requirieron hospitalización y que fallecieron. En población vacunada y con variantes circulantes menos virulentas, como la Ómicron, el SARS-CoV-2 perdió las características que habían dado lugar a la pandemia, convirtiéndose en un virus respiratorio circulante más.

El confinamiento domiciliario inicial, las medidas preventivas no farmacológicas y la vacunación han sido intervenciones eficaces y oportunas para reconducir el curso de la pandemia en diferentes momentos.
